# CRAFITY score as a predictive marker for refractoriness to atezolizumab plus bevacizumab therapy in hepatocellular carcinoma: a multicenter retrospective study

**DOI:** 10.1007/s00535-024-02150-7

**Published:** 2024-09-18

**Authors:** Masayuki Ueno, Haruhiko Takeda, Atsushi Takai, Hiroki Morimura, Norihiro Nishijima, Satoru Iwamoto, Shunsuke Okuyama, Makoto Umeda, Takeshi Seta, Atsuyuki Ikeda, Tomoyuki Goto, Shin’ichi Miyamoto, Takahisa Kayahara, Yoshito Uenoyama, Kazuyoshi Matsumura, Shigeharu Nakano, Masako Mishima, Tadashi Inuzuka, Yuji Eso, Ken Takahashi, Hiroyuki Marusawa, Yukio Osaki, Etsuro Hatano, Hiroshi Seno

**Affiliations:** 1 Department of Gastroenterology and Hepatology, Graduate School of Medicine, Kyoto University, 54 Kawahara-cho, Shogoin, Sakyo-ku, Kyoto, 606-8507 Japan; 2 Department of Gastroenterology and Hepatology, Kurashiki Central Hospital, Kurashiki, Japan; 3 Department of Gastroenterology and Hepatology, Osaka Red Cross Hospital, Osaka, Japan; 4 Department of Gastroenterology and Hepatology, Meiwa Hospital, Nishinomiya, Japan; 5 Department of Gastroenterology, Kyoto Medical Center, Kyoto, Japan; 6 Department of Gastroenterology and Hepatology, Hyogo Prefectural Amagasaki General Medical Center, Amagasaki, Japan; 7 Department of Gastroenterology and Hepatology, Japanese Red Cross Wakayama Medical Center, Wakayama, Japan; 8 Department of Health Informatics, Graduate School of Medicine and School of Public Health, Kyoto University, Kyoto, Japan; 9 Department of Gastroenterology and Hepatology, Kyoto Katsura Hospital, Kyoto, Japan; 10 Department of Medical Oncology, Shiga General Hospital, Moriyama, Japan; 11 Department of Gastroenterology and Hepatology, Shiga General Hospital, Moriyama, Japan; 12 Division of Cancer Immunotherapy, Center for Cancer Immunotherapy and Immunobiology, Graduate School of Medicine, Kyoto University, Kyoto, Japan; 13 Division of Hepato-Biliary-Pancreatic Surgery and Transplantation, Department of Surgery, Graduate School of Medicine, Kyoto University, Kyoto, Japan

**Keywords:** Liver cancer, Immune checkpoint inhibitor, C-reactive protein, Alpha-fetoprotein, Precision medicine

## Abstract

**Background:**

Although atezolizumab plus bevacizumab (Atezo/Bev) therapy has been used as the preferred first-line treatment for advanced hepatocellular carcinoma (HCC), up to 26% of patients do not achieve disease control, suggesting alternative treatments might be more beneficial for such patients. We investigated key predictors for refractoriness to Atezo/Bev therapy, particularly in the first-line setting.

**Methods:**

We retrospectively analyzed 302 patients with HCC who received Atezo/Bev therapy between October 2020 and September 2022 across nine hospitals in Japan. Refractoriness was defined as best overall response (BOR) of progressive disease or stable disease and a progression-free survival (PFS) of < 180 days (RECIST v1.1). Clinical benefit was defined as BOR of partial/complete response or stable disease with PFS of ≥ 180 days. Baseline characteristics and potential predictors, identified through literature review, were compared between these groups. Stratifications of overall survival (OS), and PFS were also assessed.

**Results:**

Refractoriness was observed in 126 (41.7%) patients, while 154 (51.0%) achieved clinical benefit. Due to a significant association between the treatment line and refractory rate, the subsequent analysis focused on the first-line cohort (*n* = 214; 72 [33.6%] patients showed refractoriness). Among 13 potential predictors, the CRP and AFP in immunotherapy (CRAFITY) score had the best predictive performance, with refractory rates of 24.6%, 44.6%, and 57.9% in CRAFITY-0, 1, and 2 patients, respectively (*p* < 0.001). OS and PFS were also well-stratified by this scoring system.

**Conclusions:**

Approximately one-third of patients were refractory to first-line Atezo/Bev therapy. The CRAFITY score demonstrated superior performance in predicting refractoriness.

**Supplementary Information:**

The online version contains supplementary material available at 10.1007/s00535-024-02150-7.

## Introduction

Hepatocellular carcinoma (HCC) ranks among the most common malignant tumors globally and stands as the third leading cause of cancer-related deaths [1]. In recent years, various molecular targeted agents have received approval for either first- [2–4] or second-line [5–7] treatment of advanced HCC. While no direct comparative trials exist for these novel drugs, current guidelines advocate for atezolizumab plus bevacizumab (Atezo/Bev) therapy as the preferred first-line option, barring contraindications [8]. This recommendation stems from data revealing longer median overall survival (OS) and a higher objective response rate (ORR) in a phase 3 clinical trial compared to other approved regimens [2–4]. However, up to 26% of patients may not achieve disease control with first-line Atezo/Bev therapy [9]. In such cases, alternative regimens like lenvatinib or durvalumab plus tremelimumab therapy could offer greater benefits. Thus, a tailored strategy for selecting the initial treatment regimen should be based on assessing the likelihood of response for each drug in each individual patient.

Several laboratory data/scoring systems have been linked to OS and/or progression-free survival (PFS) in Atezo/Bev therapy for HCC [10–13]. However, definitive evidence on predictors of poor response to this therapy is lacking. Our study aims to identify the optimal laboratory test or scoring system for predicting poor response to Atezo/Bev therapy for HCC, especially in the first-line setting. This article follows the STROBE Guidelines [14].

## Methods

### Patients

This retrospective multicenter study enrolled 302 patients with HCC who underwent Atezo/Bev therapy across nine hospitals in Japan. Inclusion criteria were: (1) histologically and/or radiologically diagnosed HCC; (2) ineligible for curative locoregional therapy (e.g., surgery, radiofrequency ablation, transcatheter arterial chemoembolization); (3) initiation of Atezo/Bev therapy between October 2020 and September 2022; and (4) age ≥ 18 years. Patients lost to follow-up within 1 month after therapy initiation were excluded. The study obtained approval from the Kyoto University Graduate School and Faculty of Medicine Ethics Committee (no. R3950) and was conducted with institutional head permission, adhering to the Declaration of Helsinki. Informed consent was obtained via an opt-out method from each patient.

### Treatment protocol

Atezo/Bev therapy was administered at standard doses, as outlined in the IMbrave 150 trial [3]. This regimen comprised intravenous administration of atezolizumab (1200 mg/body) and bevacizumab (15 mg/kg) every 3 weeks. Decisions regarding dose reduction and/or interruption were made by the treating physicians, following the protocol of the IMbrave 150 study [3]. Treatment response was evaluated at intervals of approximately two to three months using tomographic imaging tools (contrast-enhanced CT or MRI, unless contraindicated) and monthly measurements of tumor markers (α-fetoprotein [AFP] and des-gamma-carboxy prothrombin [DCP], with or without AFP-L3%). This treatment was continued until disease progression, death, or the occurrence of unacceptable adverse events. Treatment continuation beyond progression was permitted if considered acceptable by the treating physicians.

### Study endpoints

In this study, we evaluated the radiological response using the Response Evaluation Criteria in Solid Tumors version 1.1 [15]. The primary endpoint was refractoriness, which we defined as the best overall response (BOR) of progressive disease (PD) or stable disease with a PFS of < 180 days. Clinical benefit, on the other hand, was defined as either a complete or partial response or stable disease lasting ≥ 180 days, a metric sometimes referred to as the clinical benefit rate [16]. Patients who did not undergo post-treatment radiological assessment were not categorized into either the refractory or clinical benefit group. The PD rates were also assessed (Fig. S1). The ORR was defined as the proportion of patients who achieved complete or partial response, while disease control rate (DCR) was defined as the proportion of patients who achieved complete response, partial response, or stable disease. The other secondary endpoints included OS, which we defined as the duration from treatment initiation to death from any cause, and PFS, which we defined as the duration from treatment initiation until disease progression or death from any cause (cases of treatment discontinuation without progression were censored).

### Potential predictors for refractoriness

To compile potential predictive markers for refractoriness to Atezo/Bev therapy, we performed an electronic search of articles in the PubMed database published up to January 19, 2024. We identified articles pertaining to predictors for OS, PFS, ORR, and/or other efficacy indicators using the keywords "hepatocellular carcinoma," "atezolizumab," and "bevacizumab." From the reported predictive factors and scoring systems, we selected those that could be calculated using pre-treatment general laboratory data, making them applicable in real-world practice for our analysis.

### Statistical analysis

In the main analysis, baseline clinical and laboratory data of patients, alongside the aforementioned potential predictive markers, were compared between the refractory and clinical benefit groups. To examine the robustness of the findings in the main analysis, a sensitivity analysis comparing the PD and clinical benefit groups was conducted. Compared with the main analysis, the sensitivity analysis excluded patients with a stable disease and a PFS of < 180 days (Fig. S1). Continuous variables, presented as the median [interquartile range], were assessed using the Mann–Whitney *U* test. Categorical variables, expressed as numbers (percentages), underwent comparison through Fisher’s exact test. To address the effects of confounders, multivariate logistic regression analysis was conducted. The performance of predictive markers was evaluated and compared by calculating the area under the receiver operating characteristics curve (AUROC), sensitivity, specificity, and positive predictive value (PPV). Previously reported cut-off values were utilized whenever available. The DeLong test was used to compare the AUROCs of different parameters. The trend of proportions was analyzed using the Cochrane–Armitage test. Kaplan–Meier analysis, log-rank test, and Cox proportional hazard model were employed for the analysis of OS and PFS. All tests were two-tailed, and *p* values of < 0.05 were deemed statistically significant. Statistical analyses were performed using R version 4.1.2 (R Foundation).

## Results

### Patients’ characteristics

Table S1 presents an overview of the baseline characteristics of patients in both the overall and first-line cohorts. Within the entire cohort, the median age was 73 years, with 238 (78.8%) patients being male. Most patients exhibited a performance status of 0 or 1, and a Child–Pugh score of 5 or 6. A total of 214 (70.9%) patients underwent Atezo/Bev therapy as their first-line treatment for HCC, while 63 (20.9%), 17 (5.6%), 6 (2.0%), and 2 (0.7%) patients received this regimen as the second, third, fourth, and fifth-line treatments, respectively. Among patients receiving second-line or later Atezo/Bev therapy, 77 (87.5%) patients had received lenvatinib as a prior treatment. Baseline characteristics were similar between the overall and first-line cohorts. The median follow-up period was 355 days. The ORR, DCR, median PFS, and median OS were 27.5%, 79.6%, 9.5 months, and 23.0 months, respectively, in the overall cohort, and 33.3%, 85.4%, 10.6 months and 23.8 months, respectively, in the first-line cohort.

### Association between BOR and survival outcomes in the overall and first-line cohorts

**Fig. 1:**
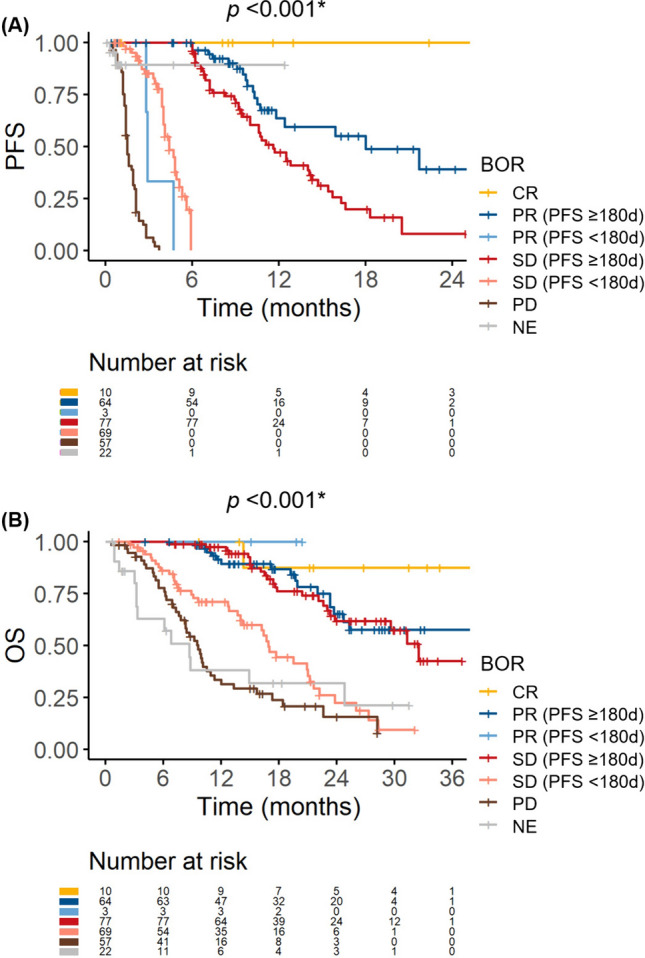
Survival outcomes stratified by best overall survival (BOR) (overall cohort). **A** Progression-free survival (PFS). **B** Overall survival (OS). In both figures, patients with a stable disease (SD) and PFS ≥ 180 days had similar survival outcomes to those with a partial response (PR). Meanwhile, patients with a SD and PFS < 180 days had similar outcomes to those with a progressive disease (PD). *CR* complete response, *NE* not evaluable

First, we examined the relationships between BOR and PFS/OS in the overall cohort. Both PFS and OS were significantly stratified based on the BOR (Fig. S2). We further observed that patients with a stable disease could be divided into two subgroups based on the PFS duration: those with a PFS of ≥ 180 days had similar PFS and OS to those with a partial response, while those with progression or censoring within 180 days had similar PFS and OS to those with a PD (Fig. 1). Thus, we opted to compare the refractory and clinical benefit groups in the main analysis (Fig. S1). Three patients exhibited a partial response and progression within 180 days. As they showed similar OS to other patients with a partial response (Fig. 1b), we did not exclude these patients from the clinical benefit group. Similar associations were observed between BOR and PFS/OS in the first-line cohort (Fig. S3).

### Refractory rate and baseline characteristics related to refractoriness in the overall cohort

In total, 126 patients (41.7%) were refractory to Atezo/Bev therapy, while 154 patients (51.0%) experienced clinical benefit. This included 10 patients with complete response and 67 with partial response (Fig. S1). The overall cohort demonstrated a PD rate of 18.9% (57/302).

**Fig. 2:**
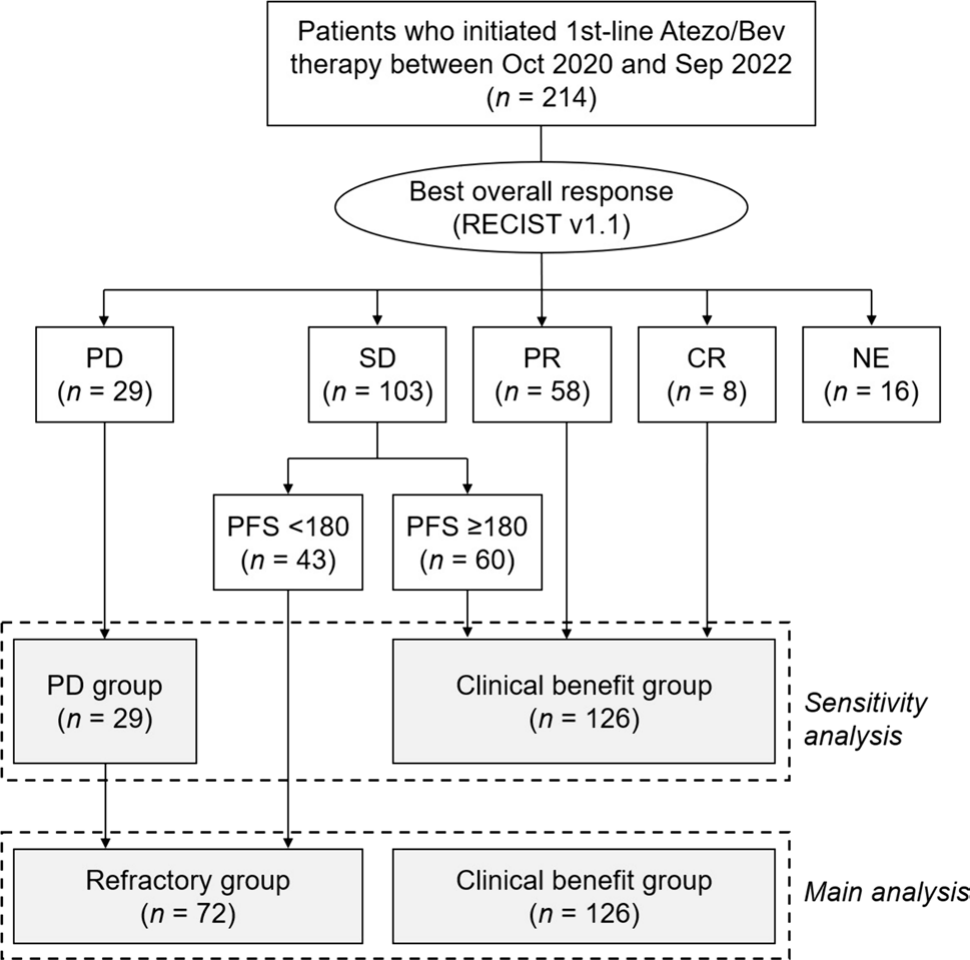
Patient allocation (1st-line cohort). Patients were classified into the refractory or clinical benefit group based on their best overall response (BOR) and progression-free survival (PFS). Patients with a progressive disease (PD) were categorized as the PD group. Some patients were not included in either group. *Atezo/Bev* atezolizumab plus bevacizumab, *SD* stable disease, *PR* partial response, *CR* complete response, *NE* not evaluable

Baseline clinical characteristics were compared between the refractory and clinical benefit groups (see Table S2). In the univariate analysis, significant differences were observed in treatment line, performance status, Child–Pugh score, albumin-bilirubin (ALBI) score, modified ALBI (mALBI) grade, Barcelona Clinic Liver Cancer (BCLC) stage, and presence/absence of macrovascular invasion. As the second or later line of treatment showed a significant association with a higher refractory rate (54/82, 65.9% vs. 72/198, 36.4%; *p* < 0.001), and our focus was on identifying predictors for poor response to first-line Atezo/Bev therapy, we restricted the study population for subsequent analysis to those who received this therapy as their first-line treatment. In the first-line cohort, 72 patients (33.6%) were refractory to Atezo/Bev therapy, while 126 patients (58.9%) experienced clinical benefit (Fig. 2). The first-line cohort exhibited a PD rate of 13.6% (29/214).

### Literature review of potential predictors for refractoriness

The results of the literature review are summarized in Table S3. The most validated marker was the neutrophil-to-lymphocyte ratio (NLR), originally reported from one of our affiliated institutions [10]. However, the associated cut-off values differed among studies. The second most validated marker was the (modified) ALBI grade/score, which was first reported by de Castro et al. [17]. The C-reactive protein (CRP) and AFP in immunotherapy (CRAFITY) score, in which the variables AFP ≥ 100 ng/mL and CRP ≥ 1 mg/dL are each assigned 1 point, was initially reported to be associated with the efficacy of programmed death 1 (PD-1)- or programmed death ligand 1 (PD-L1)-based immunotherapy [18]. Its association with the efficacy of Atezo/Bev therapy was subsequently reported by three groups [11, 19, 20]. Other predictive markers had either no or a limited number of validation reports. Among the extracted scoring systems, the CRAFITY score, modified ALBI grade and AFP (mALF) score, neo-Glasgow prognostic score, Atezo/Bev index, and geriatric nutritional risk index could be calculated without missing data in this study.

### Predictors for refractoriness in the first-line cohort

In the first-line cohort, worse performance status, Child–Pugh score, ALBI scores, and mALBI grade, advanced BCLC stage, presence of macrovascular invasion, and certain laboratory data including CRP, AFP, and DCP were significantly associated with a higher refractory rate (Table 1). The performance of each potential predictor identified through the literature review is summarized in Table 2. Continuous markers tended to exhibit a good AUROC; however, their predictive values with a specific cut-off value were not sufficient. Among categorical predictors, both the mALF score and CRAFITY score demonstrated a good AUROC and a high PPV. Although the difference in the AUROCs of these two parameters in predicting refractoriness was not statistically significant (*p* = 0.142) (Figure S4A), a multivariate analysis using the Cox proportional hazard model identified a CRP of ≥ 1.0 mg/dL (a component of the CRAFITY scoring system) as the most significant factor for PFS, compared with mALBI grade 2b/3 (a component of the mALF scoring system) and an AFP of ≥ 100 ng/mL (a component of both systems) (Table S4). Therefore, the CRAFITY score was the optimal method for predicting poor responders to first-line Atezo/Bev therapy. Nonetheless, a multivariate analysis including the CRAFITY score, performance status, mALBI grade, BCLC stage, and macrovascular invasion revealed that both CRAFITY score and mALBI grade 2b/3 were independently and significantly associated with refractoriness in the first-line setting (Table 3). In addition, subgroup analyses consistently showed a negative impact of a CRAFITY score of 2 on the risk of refractoriness (Table S5).

**Table 1:** Comparison of the baseline characteristics between the refractory and clinical benefit groups in the first-line cohort

	Refractory (*n* = 72)	Clinical benefit (*n* = 126)	*p* value
Age, years	73 [66.8–79]	73 [68–78]	0.976
Sex			0.234
Male	50 (69.4%)	98 (77.8%)	
Female	22 (30.6%)	28 (22.2%)	
Performance status			0.025*
0	51 (70.8%)	106 (84.1%)	
1	18 (25.0%)	18 (14.3%)	
2	3 (4.2%)	2 (1.6%)	
Body mass index, kg/m^2^	23.6 [21.2–25.7]	24.1 [21.2–26.3]	0.437
Etiology of liver diseases			0.768
Viral	35 (48.6%)	58 (46.0%)	
Non-viral	37 (51.4%)	68 (54.0%)	
Steatotic liver disease			0.443
Presence	30 (41.7%)	45 (35.7%)	
Absence	39 (54.2%)	75 (59.5%)	
Unknown	3 (4.2%)	6 (4.8%)	
Child–Pugh score			0.009*
5	30 (41.7%)	74 (58.7%)	
6	30 (41.7%)	43 (34.1%)	
7	9 (12.5%)	8 (6.3%)	
8	3 (4.2%)	1 (0.8%)	
ALBI score	− 2.20 [− 2.60 to − 1.96]	− 2.53 [− 2.73 to − 2.28]	0.001*
mALBI grade			< 0.001*
1	18 (25.0%)	56 (44.4%)	
2a	17 (23.6%)	40 (31.7%)	
2b	36 (50.0%)	30 (23.8%)	
3	1 (1.4%)	0 (0%)	
BCLC stage			0.006*
Early stage	6 (8.3%)	9 (7.1%)	
Intermediate stage	24 (33.3%)	73 (57.9%)	
Advanced stage	42 (58.3%)	44 (34.9%)	
Macrovascular invasion	21 (29.2%)	14 (11.1%)	0.002*
Extrahepatic metastasis	26 (36.1%)	34 (27.0%)	0.200
NLR	2.7 [2.1–4.1]	2.3 [1.7–3.1]	0.026*
PLR	122 [93–169]	108 [83–155]	0.118
CRP, mg/dL	0.3 [0.2–0.8]	0.2 [0.1–0.3]	< 0.001*
AFP, ng/mL	95 [8–1879]	8 [4–59]	< 0.001*
DCP, mAU/mL	843 [115–8805]	213 [49–2470]	0.006*

Data are expressed as number (percentage) or median [interquartile range]

*AFP* α-fetoprotein, *ALBI* albumin-bilirubin, *BCLC* Barcelona Clinical Liver Cancer, *CRP* C-reactive protein, *DCP* des-gamma-carboxy prothrombin, *mALBI* modified albumin-bilirubin, *NLR* neutrophil-to-lymphocyte ratio, *PLR* platelet-to-lymphocyte ratio

*p < 0.05

**Table 2:** Performance of potential predictors for refractoriness to atezolizumab plus bevacizumab therapy in the first-line setting (main analysis cohort)

Predictors	AUROC (95% CI)	Cut-off	Sensitivity (%)	Specificity (%)	PPV
Continuous values					
CRP	0.673 (0.594–0.753)	≥ 1.0	20.8	92.1	15/25 (60.0%)
AFP	0.688 (0.610–0.767)	≥ 400	34.7	84.9	25/44 (56.8%)
DCP	0.617 (0.535–0.699)	≥ 400	61.1	60.3	44/94 (46.8%)
NLR	0.595 (0.511–0.680)	≥ 3.21	40.3	76.2	29/59 (49.2%)
PLR	0.567 (0.483–0.650)	≥ 230	13.9	90.5	10/22 (45.5%)
ALBI score	0.639 (0.555–0.722)	≥ − 2.3	52.8	73.8	38/71 (53.5%)
PNI	0.647 (0.567–0.727)	≤ 47	80.6	32.5	58/143 (40.6%)
Categorical values					
mALBI grade	0.652 (0.576–0.729)	1/2a vs. 2b/3	51.4	76.2	37/67 (55.2%)
CRAFITY score	0.657 (0.586–0.728)	0/1 vs. 2	15.3	97.6	11/14 (78.6%)
mALF score	0.700 (0.629–0.772)	0/1 vs. 2	29.2	93.7	21/29 (72.4%)
Neo-GPS	0.630 (0.559–0.701)	0 vs. 1/2	77.8	42.9	56 128 (43.8%)
ABE index	0.602 (0.537–0.667)	Low/intermediate vs. high-risk	76.4	42.1	55/128 (43.0%)
GNRI	0.598 (0.522–0.674)	Normal/mild vs. moderate/severe	30.6	87.3	22/38 (57.9%)

This cohort includes patients with a stable disease and a progression-free survival of < 180 days

*ABE* atezolizumab plus bevacizumab, *AFP* α-fetoprotein, *ALBI* albumin-bilirubin, *AUROC* area under the receiver operating characteristics curve, *CRAFITY* CRP and *AFP* in immunotherapy, *CRP* C-reactive protein, *DCP* des-gamma-carboxy prothrombin, *GNRI* geriatric nutritional risk index, *mALBI* modified albumin-bilirubin, *mALF* modified ALBI grade and AFP, *neo-GPS* neo-Glasgow prognostic score, *NLR* neutrophil-to-lymphocyte ratio, *PLR* platelet-to-lymphocyte ratio, *PNI* prognostic nutritional index, *PPV* positive predictive value

**Table 3:** Multivariate analysis of the predictors for refractoriness in the first-line setting (main analysis cohort)

Variables	Odds ratio	95% CI	*p* value
Performance status			
0	1 (reference)		
1/2	1.68	0.77–3.67	0.192
BCLC stage			
Early stage	1 (reference)		
Intermediate stage	0.53	0.15–1.86	0.324
Advanced stage	1.10	0.30–4.05	0.881
Macrovascular invasion			
Absent	1 (reference)		
Present	1.42	0.53–3.81	0.482
CRAFITY score			
0	1 (reference)		
1	2.31	1.16–4.61	0.017*
2	5.03	1.17–21.7	0.030*
mALBI grade			
1	1 (reference)		
2a	1.51	0.64–3.56	0.346
2b/3	3.12	1.44–6.74	0.004*

This cohort includes patients with a stable disease and a progression-free survival of < 180 days

*BCLC* Barcelona Clinical Liver Cancer, *CI* confidence interval, *CRAFITY* C-reactive protein and α-fetoprotein in immunotherapy, *mALBI* modified albumin-bilirubin

*p < 0.05

As a sensitivity analysis, we compared the baseline characteristics between the PD and clinical benefit groups. Similar to the main analysis, the PD group had significantly worse ALBI score and mALBI grade, more advanced BCLC stage, higher frequency of macrovascular invasion, and higher levels of CRP, AFP, and DCP compared with the clinical benefit group (Table S6). The performance of each potential predictor in distinguishing between these two groups is summarized in Table S7. The CRP and AFP levels showed relatively robust AUROC values among the continuous variables, and the CRAFITY score demonstrated the highest PPV among all predictors. The AUROC values of the CRAFITY and mALF scores were not significantly different (*p* = 0.549) (Fig. S4B). These results supported the robustness of CRAFITY score’s high performance in predicting poor response to first-line Atezo/Bev therapy.

### Associations between the CRAFITY score with BOR, OS and PFS

**Fig. 3:**
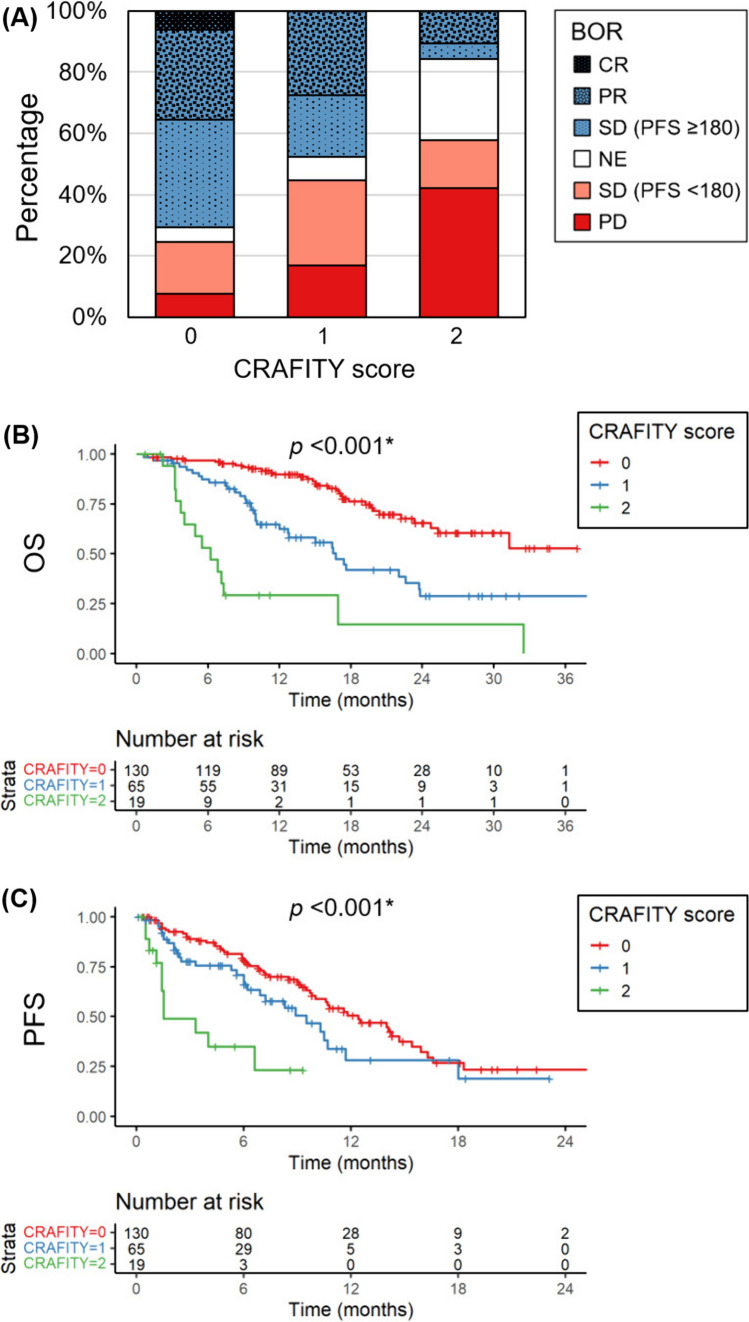
Associations between the CRAFITY score and treatment outcomes with first-line atezolizumab plus bevacizumab therapy. **A** Best overall response (BOR) in patients stratified by CRAFITY score. Red and blue columns represent the refractory and clinical benefit groups, respectively. *CR* complete response, *PR* partial response, *SD* stable disease, *PFS* progression-free survival, *NE* not evaluable, *PD* progressive disease. **B** Overall survival (OS) stratified with the CRAFITY score. **C** PFS stratified with the CRAFITY score

Finally, we investigated the impact of the CRAFITY score on BOR, OS, and PFS; in this analysis, all patients were analyzed irrespective of their BOR or PFS. In the first-line setting, the refractory rate significantly increased along with the CRAFITY score, with rates of 24.6%, 44.6%, and 57.9% in patients with CRAFITY-0, 1, and 2, respectively (*p* < 0.001) (Fig. 3A). The PD rates were 7.7%, 16.9%, and 42.1% in patients with CRAFITY-0, 1, and 2, respectively (*p* < 0.001). Complete response was only achieved in CRAFITY-0 patients. The CRAFITY score significantly stratified OS and PFS. The median OS was not reached, 16.7 months, and 6.2 months in patients with CRAFITY-0, 1, and 2, respectively (*p* < 0.001) (Fig. 3B). Compared to CRAFITY-0 patients, hazard ratios of OS in CRAFITY-1 and 2 patients were 2.86 (95% confidence interval, 1.76–4.64) and 8.83 (4.63–16.86), respectively. The median PFS was 12.4 months, 9.5 months, and 1.5 months in patients with CRAFITY-0, 1, and 2, respectively (*p* < 0.001) (Fig. 3C). Hazard ratios of PFS in CRAFITY-1 and 2 patients were 1.49 (0.95–2.34) and 5.00 (2.55–9.81), respectively.

**Fig. 4:**
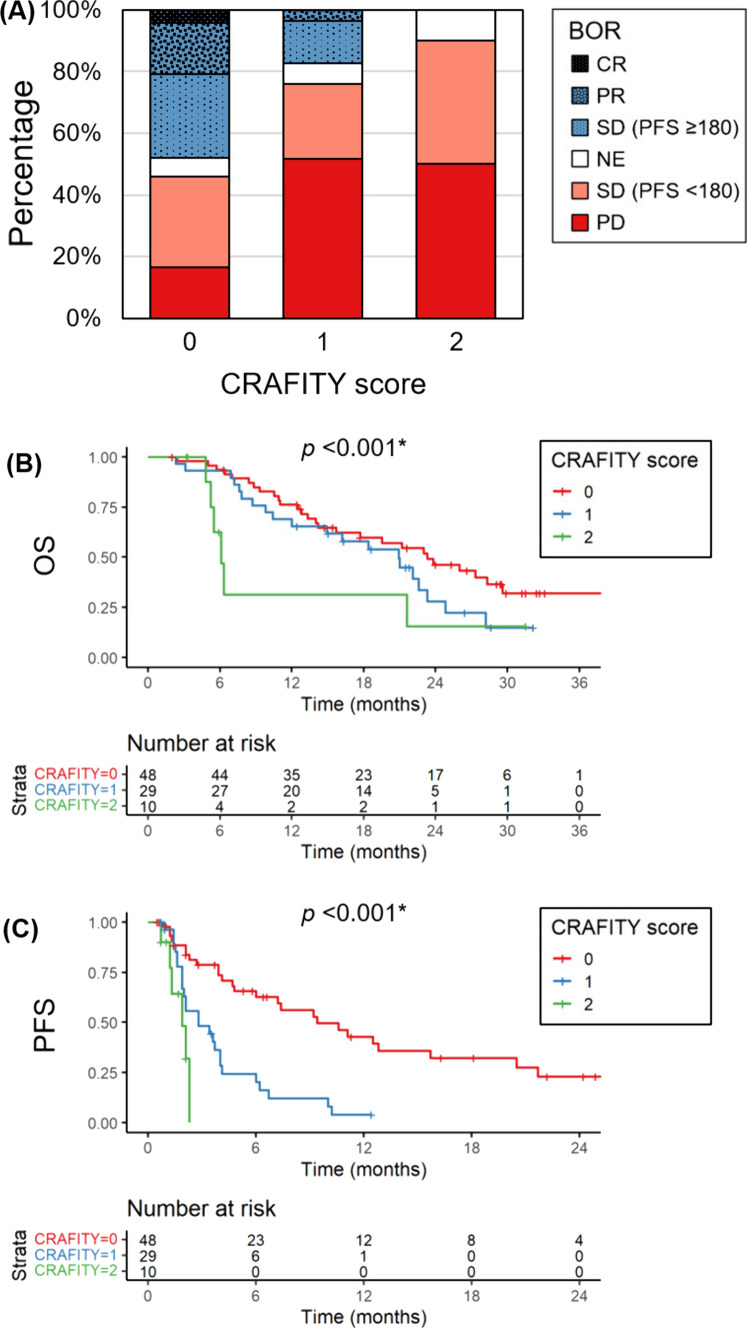
Associations between the CRAFITY score and treatment outcomes with second- or later line atezolizumab plus bevacizumab therapy. **A** Best overall response (BOR) in patients stratified by the CRAFITY score. Red and blue columns represent the refractory and clinical benefit groups, respectively. *CR* complete response, *PR* partial response, *SD* stable disease, *PFS* progression-free survival, *NE* not evaluable, *PD* progressive disease. **B** Overall survival (OS) stratified with the CRAFITY score. **C** PFS stratified with the CRAFITY score

Similarly, in the second- or later-line setting, the refractory rates significantly increased along with the CRAFITY score, with rates of 45.8%, 75.9%, and 90.0% in patients with CRAFITY-0, 1, and 2, respectively (*p* = 0.001) (Fig. 4A). The PD rates were 16.7%, 51.7%, and 50.0% in patients with CRAFITY-0, 1, and 2, respectively (*p* = 0.002). The median OS was 23.3 months, 20.9 months, and 6.1 months in patients with CRAFITY-0, 1, and 2, respectively (*p* < 0.001) (Fig. 4B). The median PFS was 9.4 months, 2.8 months, and 1.9 months in patients with CRAFITY-0, 1, and 2, respectively (*p* < 0.001) (Fig. 4C).

## Discussion

The present study underscores the superior predictive performance of the CRAFITY score compared to other markers in anticipating refractoriness to Atezo/Bev therapy in HCC patients, both in first- and later line settings. Noteworthy aspects of this research include prioritizing the refractory and PD rates in assessing treatment efficacy and conducting a comprehensive comparison of all available markers reported in previous literature.

Numerous reports on predictors for the efficacy of Atezo/Bev therapy were identified through systematic review. Most of the studies retrieved from the literature focused on OS, PFS, and/or ORR in evaluating treatment efficacy. However, since this regimen is predominantly used as first-line treatment in current practice, predicting favorable responders has limited impact on altering treatment strategy. Nonetheless, anticipating poor responders is valuable as it can guide the preference for other regimens in these patients. Intriguingly, NLR, which has been most frequently reported to be associated with OS and PFS [10, 12, 21–29], showed relatively lower performance in predicting refractoriness. Thus, it is important to further distinguish predictors for refractoriness and those for survival outcomes. Moreover, we evaluated the PD rate based on the BOR, instead of early PD in previous studies [30, 31], because pseudoprogression is sometimes experienced in immunotherapy [32]. Patients with a stable disease showed various prognoses. Hence, both ORR (which categorizes all stable patients as non-responders) and DCR (which categorizes these patients as responders) were deemed inappropriate indicators for distinguishing between favorable responders and poor responders. Thus, we compared patients who exhibited refractoriness and those with clinical benefit, based on the reports of previous studies on other cancer types [33, 34]. Our study highlights the usefulness of the CRAFITY score in identifying patients unlikely to respond to first-line Atezo/Bev therapy, thereby facilitating a tailored treatment strategy for advanced HCC.

Despite significant efforts to explore predictive markers for the efficacy of Atezo/Bev therapy to date, none have been utilized reliably to influence the choice of the first-line regimen for HCC. This is largely due to most markers not being validated by other studies using the same cut-off values. For instance, a large-scale multicenter study group in Japan has reported nine predictive markers associated with OS and/or PFS [11, 21, 29, 35–40], but most of them have not been validated by other researchers. Our study is the first large-scale study to compare the predictive performance of these previously reported markers, revealing that the CRAFITY score was superior to other scoring systems for predicting refractoriness.

The CRAFITY score was initially reported by Scheiner et al. [18], who analyzed 292 HCC patients receiving PD-(L)1-based immunotherapy at any line of systemic therapy. They found that CRP ≥ 1 mg/dL and AFP ≥ 100 ng/mL were independently and significantly associated with OS. Although the mechanism of refractoriness to Atezo/Bev therapy has not been fully understood, both tumor biology and the tumor microenvironment play important roles [41]. CRP is a well-accepted marker of cancer-induced inflammation, associated with cancer cell proliferation, angiogenesis, and the inhibition of adaptive immunity [42]. AFP is the most widely used tumor marker for HCC, which is known to be related to tumor growth and antitumor immunity [43]. Ramucirumab, an anti-vascular endothelial growth factor agent, has been shown to be effective in AFP-high HCC; however, even when treated with this drug, higher AFP levels are associated with worse OS [44]. Thus, it is reasonable that CRP and AFP are associated with refractoriness to Atezo/Bev therapy.

Recently, combinations of the CRAFITY score with other clinical or radiological parameters have been reported [45, 46]. However, calculating these complex scoring systems requires assessing sarcopenia and interpreting MRI findings. In contrast, our study suggests that incorporating treatment line and pre-treatment liver function may improve predictive accuracy without extending calculation time. Although the combination of the CRAFITY score and AFP response has been reported [19, 47], it cannot guide initial treatment decisions because AFP response can only be evaluated after starting Atezo/Bev therapy.

Our study has several limitations. Firstly, being a retrospective study, the timing and intervals of radiological assessments may have varied among treating physicians. Prospective studies are needed to validate the utility of the CRAFITY score in predicting refractoriness to Atezo/Bev therapy. Secondly, some of the previously reported predictive markers could not be calculated due to missing data. Thirdly, we did not examine the impact of the CRAFITY score on the efficacy of other regimens in this study. However, Scheiner et al. reported that the CRAFITY score was not associated with the disease control rate with sorafenib [18]; thus, tyrosine kinase inhibitors may be effective in patients with a high CRAFITY score. Further studies should explore whether lenvatinib or durvalumab plus tremelimumab therapy demonstrates superior efficacy to Atezo/Bev therapy in CRAFITY-1 or 2 patients.

In conclusion, our comparison of various reported markers revealed the CRAFITY score as the most reliable predictor for refractoriness to first-line Atezo/Bev therapy. Additionally, this parameter could effectively stratify the PD rate, BOR, OS, and PFS. Our findings will contribute to the development of a tailored treatment strategy for advanced HCC.

## Supplementary Information

Below is the link to the electronic supplementary material.

Supplementary file1 Fig. S1. Patient allocation (overall cohort). Patients were classified into the refractory or clinical benefit group based on their best overall response (BOR) and progression-free survival (PFS). Patients with a progressive disease (PD) were categorized as the PD group. Some patients were not included in either group. Atezo/Bev, atezolizumab plus bevacizumab; SD, stable disease; PR, partial response; CR, complete response; NE, not evaluable (TIF 136 KB)

Supplementary file2 Fig. S2. Survival outcomes stratified based on the best overall response (BOR) (overall cohort). (A) The progression-free survival (PFS) was clearly stratified based on the BOR. (B) Overall survival (OS) was also stratified based on the BOR. PD, progressive disease; SD, stable disease; PR, partial response; CR, complete response; NE, not evaluable (TIF 319 KB)

Supplementary file3 Fig. S3. Survival outcomes stratified based on the best overall response (BOR) (first-line cohort). (A) Progression-free survival (PFS). (B) Overall survival (OS). In both figures, the patients with a stable disease (SD) and PFS of ≥180 days had similar survival outcomes to those with a partial response (PR). Meanwhile, patients with a SD and PFS < 180 days had similar outcomes to those with a progressive disease (PD). CR, complete response; NE, not evaluable (TIF 360 KB)

Supplementary file4 Fig. S4. Comparison of the receiver operating characteristics curves of the CRAFITY and mALF scores. No significant differences were observed in the performance of the CRAFITY and mALF scores in predicting the refractoriness to atezolizumab plus bevacizumab therapy, both in (A) the first-line (p = 0.142) and (B) second- or later-line settings (p = 0.058) (TIF 113 KB)

Supplementary file5 (DOCX 135 KB)
