# Publisher Correction: CRAFITY score as a predictive marker for refractoriness to atezolizumab plus bevacizumab therapy in hepatocellular carcinoma: a multicenter retrospective study

**DOI:** 10.1007/s00535-024-02159-y

**Published:** 2024-10-21

**Authors:** Masayuki Ueno, Haruhiko Takeda, Atsushi Takai, Hiroki Morimura, Norihiro Nishijima, Satoru Iwamoto, Shunsuke Okuyama, Makoto Umeda, Takeshi Seta, Atsuyuki Ikeda, Tomoyuki Goto, Shin’ichi Miyamoto, Takahisa Kayahara, Yoshito Uenoyama, Kazuyoshi Matsumura, Shigeharu Nakano, Masako Mishima, Tadashi Inuzuka, Yuji Eso, Ken Takahashi, Hiroyuki Marusawa, Yukio Osaki, Etsuro Hatano, Hiroshi Seno

**Affiliations:** 1 Department of Gastroenterology and Hepatology, Graduate School of Medicine, Kyoto University, 54 Kawahara-cho, Shogoin, Sakyo-ku, Kyoto, 606-8507 Japan; 2 Department of Gastroenterology and Hepatology, Kurashiki Central Hospital, Kurashiki, Japan; 3 Department of Gastroenterology and Hepatology, Osaka Red Cross Hospital, Osaka, Japan; 4 Department of Gastroenterology and Hepatology, Meiwa Hospital, Nishinomiya, Japan; 5 Department of Gastroenterology, Kyoto Medical Center, Kyoto, Japan; 6 Department of Gastroenterology and Hepatology, Hyogo Prefectural Amagasaki General Medical Center, Amagasaki, Japan; 7 Department of Gastroenterology and Hepatology, Japanese Red Cross Wakayama Medical Center, Wakayama, Japan; 8 Department of Health Informatics, Graduate School of Medicine and School of Public Health, Kyoto University, Kyoto, Japan; 9 Department of Gastroenterology and Hepatology, Kyoto Katsura Hospital, Kyoto, Japan; 10 Department of Medical Oncology, Shiga General Hospital, Moriyama, Japan; 11 Department of Gastroenterology and Hepatology, Shiga General Hospital, Moriyama, Japan; 12 Division of Cancer Immunotherapy, Center for Cancer Immunotherapy and Immunobiology, Graduate School of Medicine, Kyoto University, Kyoto, Japan; 13 Division of Hepato-Biliary-Pancreatic Surgery and Transplantation, Department of Surgery, Graduate School of Medicine, Kyoto University, Kyoto, Japan

**Publisher Correction: Journal of Gastroenterology** 10.1007/s00535-024-02150-7

In the sentence beginning 'Despite significant efforts to explore predictive…' in this article, the reference citation '[11, 21, 35–4029]' should have read '[11, 21, 29, 35–40]'. In HTML version, it should have read ‘[11, 21, 29, 35, 36, 37, 38, 39, 40]’. The publisher sincerely apologizes for any inconvenience these errors may have caused.

The original article has been corrected.

