# Supplementary figures and images for: CRAFITY score as a predictive marker for refractoriness to atezolizumab plus bevacizumab therapy in hepatocellular carcinoma: a multicenter retrospective study
Source: J Gastroenterol. 2024 Sep 18;59(12):1107–18. doi: 10.1007/s00535-024-02150-7 (PMC11541291; doi:10.1007/s00535-024-02150-7)

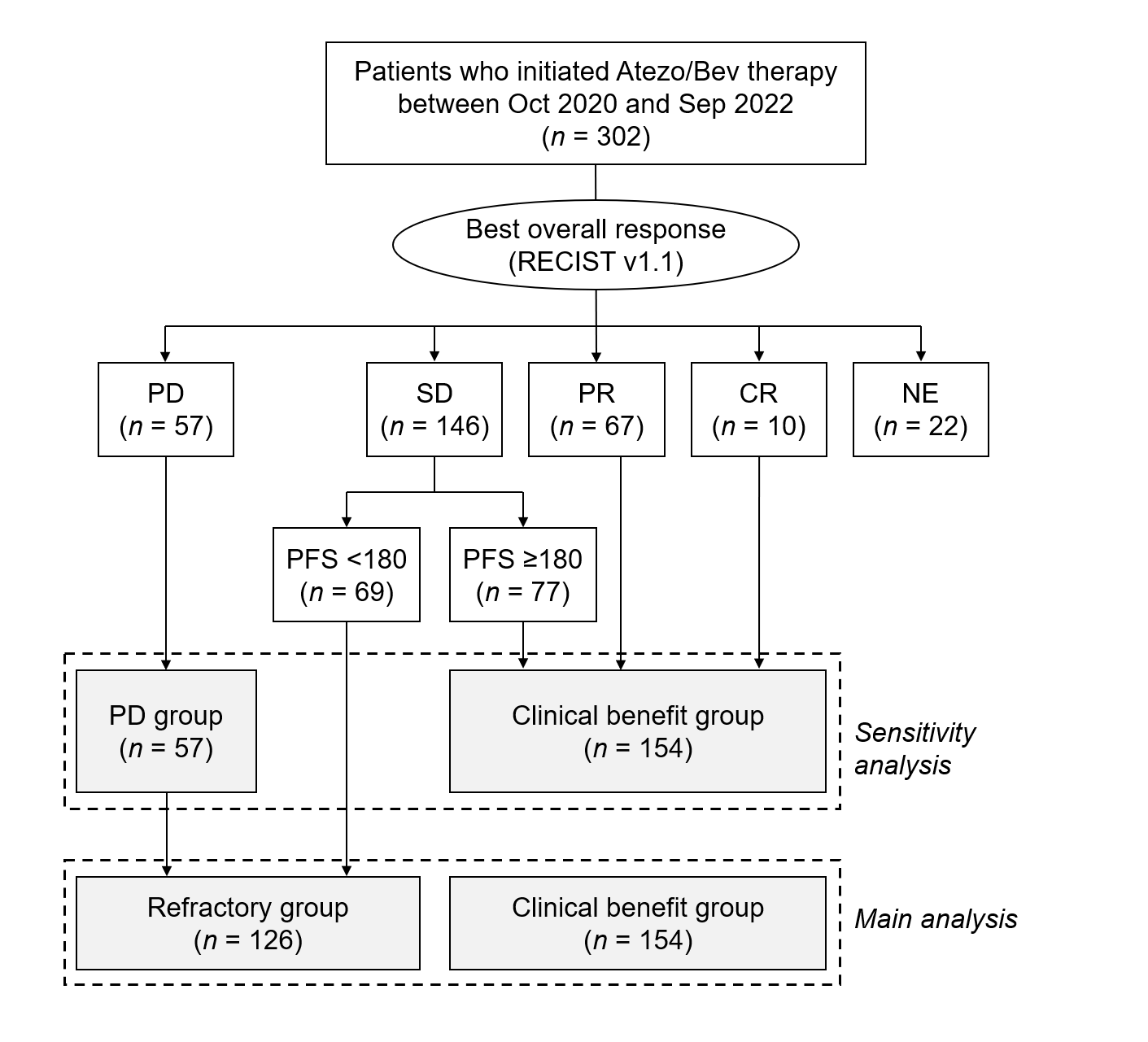

Supplement: Supplementary file 1 — Supplementary file1 Fig. S1. Patient allocation (overall cohort). Patients were classified into the refractory or clinical benefit group based on their best overall response (BOR) and progression-free survival (PFS). Patients with a progressive disease (PD) were categorized as the PD group. Some patients were not included in either group. Atezo/Bev, atezolizumab plus bevacizumab; SD, stable disease; PR, partial response; CR, complete response; NE, not evaluable (TIF 136 KB) [file 535_2024_2150_MOESM1_ESM.tif]

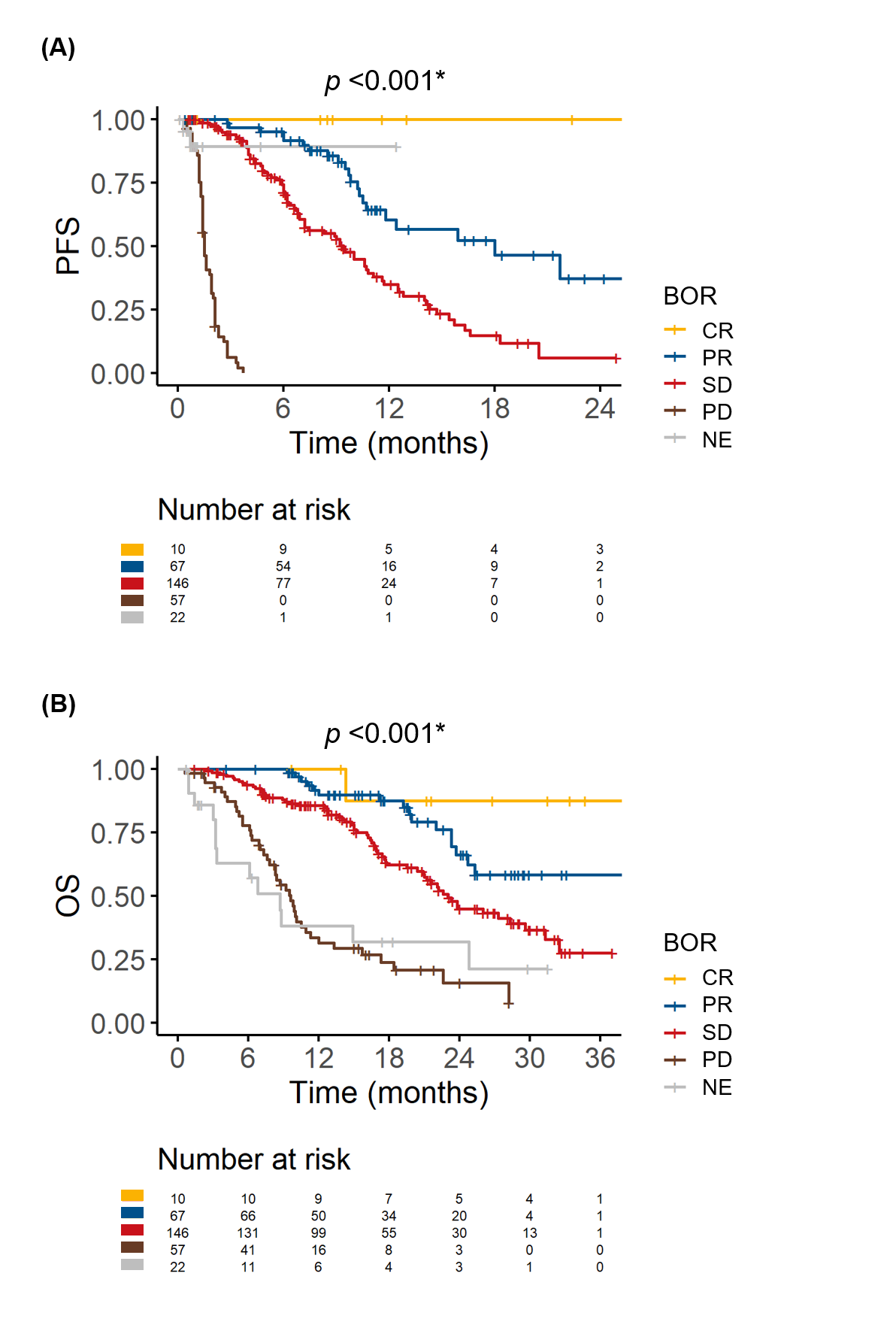

Supplement: Supplementary file 2 — Supplementary file2 Fig. S2. Survival outcomes stratified based on the best overall response (BOR) (overall cohort). (A) The progression-free survival (PFS) was clearly stratified based on the BOR. (B) Overall survival (OS) was also stratified based on the BOR. PD, progressive disease; SD, stable disease; PR, partial response; CR, complete response; NE, not evaluable (TIF 319 KB) [file 535_2024_2150_MOESM2_ESM.tif]

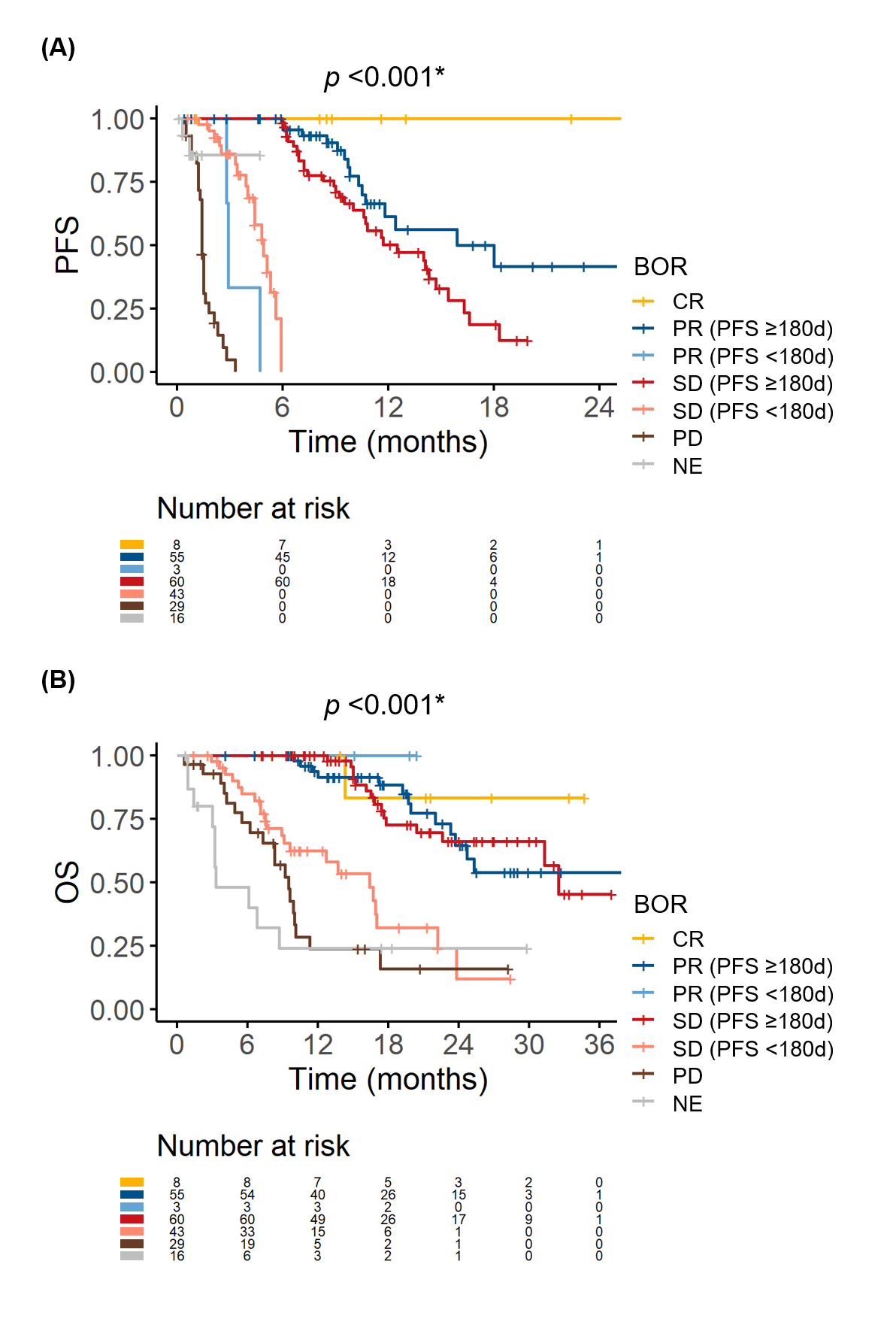

Supplement: Supplementary file 3 — Supplementary file3 Fig. S3. Survival outcomes stratified based on the best overall response (BOR) (first-line cohort). (A) Progression-free survival (PFS). (B) Overall survival (OS). In both figures, the patients with a stable disease (SD) and PFS of ≥180 days had similar survival outcomes to those with a partial response (PR). Meanwhile, patients with a SD and PFS < 180 days had similar outcomes to those with a progressive disease (PD). CR, complete response; NE, not evaluable (TIF 360 KB) [file 535_2024_2150_MOESM3_ESM.tif]

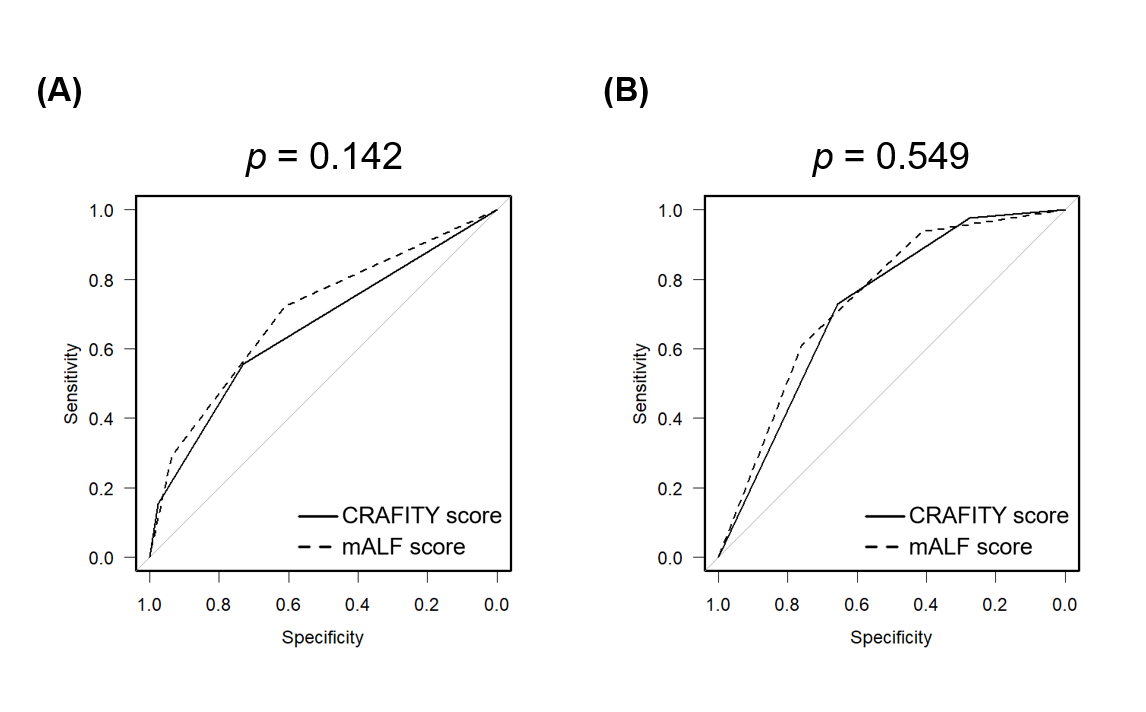

Supplement: Supplementary file 4 — Supplementary file4 Fig. S4. Comparison of the receiver operating characteristics curves of the CRAFITY and mALF scores. No significant differences were observed in the performance of the CRAFITY and mALF scores in predicting the refractoriness to atezolizumab plus bevacizumab therapy, both in (A) the first-line (p = 0.142) and (B) second- or later-line settings (p = 0.058) (TIF 113 KB) [file 535_2024_2150_MOESM4_ESM.tif]
