# Supplementary material for: CRAFITY score as a predictive marker for refractoriness to atezolizumab plus bevacizumab therapy in hepatocellular carcinoma: a multicenter retrospective study
Source: J Gastroenterol. 2024 Sep 18;59(12):1107–18. doi: 10.1007/s00535-024-02150-7 (PMC11541291; doi:10.1007/s00535-024-02150-7)
Supplement: Supplementary file 5 — Supplementary file5 (DOCX 135 KB) [file 535_2024_2150_MOESM5_ESM.docx]

**Table S1. Characteristics of the study participants**

|  | All patients  (*n* = 302) | 1st-line  (*n* = 214) |
| --- | --- | --- |
| Age, years | 73 [66–79] | 73 [67–79] |
| Sex |  |  |
| Male | 238 (78.8%) | 160 (74.8%) |
| Female | 64 (21.2%) | 54 (25.2%) |
| Performance status |  |  |
| 0 | 224 (74.2%) | 168 (78.5%) |
| 1 | 72 (23.8%) | 40 (18.7%) |
| 2 | 6 (2.0%) | 6 (2.8%) |
| Body mass index, kg/m^2^ | 23.6 [21.0–25.8] | 23.7 [21.1–26.1] |
| Etiology of liver diseases |  |  |
| HBV | 57 (18.9%) | 37 (17.3%) |
| HCV | 86 (28.5%) | 60 (28.0%) |
| ALD | 69 (22.9%) | 51 (23.8%) |
| MASLD | 59 (19.5%) | 43 (20.1%) |
| HBV and HCV | 1 (0.3%) | 1 (0.5%) |
| HBV/HCV and ALD | 7 (2.3%) | 5 (2.3%) |
| Others | 23 (7.6%) | 17 (7.9%) |
| Steatotic liver disease |  |  |
| Presence | 110 (36.4%) | 79 (36.9%) |
| Absence | 177 (58.6%) | 125 (58.4%) |
| Unknown | 15 (5.0%) | 10 (4.7%) |
| Child–Pugh score |  |  |
| 5 | 152 (50.3%) | 113 (52.8%) |
| 6 | 106 (35.1%) | 77 (36.0%) |
| 7 | 37 (12.3%) | 20 (9.4%) |
| 8 | 7 (2.3%) | 4 (1.9%) |
| ALBI score | -2.43 [-2.69 to -2.05] | -2.48 [-2.70 to -2.08] |
| mALBI grade |  |  |
| 1 | 103 (34.1%) | 76 (35.5%) |
| 2a | 85 (28.2%) | 63 (29.4%) |
| 2b | 110 (36.4%) | 74 (34.6%) |
| 3 | 4 (1.3%) | 1 (0.5%) |
| BCLC stage |  |  |
| Early stage | 23 (7.6%) | 17 (7.9%) |
| Intermediate stage | 142 (47.0%) | 103 (48.1%) |
| Advanced stage | 137 (45.4%) | 94 (43.9%) |
| Macrovascular invasion | 54 (17.9%) | 39 (18.2%) |
| Extrahepatic metastasis | 100 (33.1%) | 65 (30.4%) |
| AFP, ng/mL | 19 [5–283] | 16 [5–283] |
| DCP, mAU/mL | 480 [66–4857] | 395 [61–4212] |

AFP, α-fetoprotein; ALBI, albumin-bilirubin; ALD, alcoholic liver disease; BCLC, Barcelona Clinical Liver Cancer; DCP, des-gamma-carboxy prothrombin; HBV, hepatitis B virus; HCV, hepatitis C virus; mALBI, modifed albumin-bilirubin; MASLD, metabolic dysfunction-associated steatotic liver disease.

Data are expressed as number (percentage) or median [interquartile range].

**Table S2. Comparison of baseline characteristics between the refractory and clinical benefit groups in the overall cohort**

|  | Refractory  (*n* = 126) | Clinical benefit  (*n* = 154) | *p* value |
| --- | --- | --- | --- |
| Treatment line |  |  | <0.001* |
| 1st-line | 72 (57.1%) | 126 (81.8%) |  |
| 2nd-line or later | 54 (42.9%) | 28 (18.2%) |  |
| Age, years | 72 [66–78] | 73 [68–78] | 0.271 |
| Sex |  |  | 0.769 |
| Male | 98 (77.8%) | 123 (79.9%) |  |
| Female | 28 (22.2%) | 31 (20.1%) |  |
| Performance status |  |  | 0.013* |
| 0 | 87 (69.0%) | 126 (81.8%) |  |
| 1 | 36 (28.6%) | 26 (16.9%) |  |
| 2 | 3 (2.4%) | 2 (1.3%) |  |
| Body mass index, kg/m^2^ | 23.6 [21.2–25.0] | 23.8 [21.1–26.1] | 0.335 |
| Etiology of liver diseases |  |  | 0.548 |
| Viral | 64 (50.8%) | 72 (46.8%) |  |
| Non-viral | 62 (49.2%) | 82 (53.2%) |  |
| Steatotic liver disease |  |  | 0.530 |
| Presence | 49 (38.9%) | 55 (35.7%) |  |
| Absence | 70 (55.6%) | 93 (60.4%) |  |
| Unknown | 7 (5.6%) | 6 (3.9%) |  |
| Child–Pugh score |  |  | 0.004* |
| 5 | 56 (44.4%) | 87 (56.5%) |  |
| 6 | 42 (33.3%) | 56 (36.4%) |  |
| 7 | 22 (17.5%) | 10 (6.5%) |  |
| 8 | 6 (4.8%) | 1 (0.6%) |  |
| ALBI score | -2.28 [-2.61 to -1.94] | -2.53 [-2.73 to -2.22] | <0.001* |
| mALBI grade |  |  | <0.001* |
| 1 | 34 (27.0%) | 67 (43.5%) |  |
| 2a | 30 (23.8%) | 46 (29.9%) |  |
| 2b | 58 (46.0%) | 41 (26.6%) |  |
| 3 | 4 (3.2%) | 0 (0%) |  |
| BCLC stage |  |  | 0.025* |
| Early stage | 10 (7.9%) | 11 (7.1%) |  |
| Intermediate stage | 49 (38.9%) | 85 (55.2%) |  |
| Advanced stage | 67 (53.2%) | 58 (37.7%) |  |
| Macrovascular invasion | 33 (26.2%) | 17 (11.0%) | 0.002* |
| Extrahepatic metastasis | 45 (35.7%) | 46 (29.9%) | 0.308 |

ALBI, albumin-bilirubin; BCLC, Barcelona Clinical Liver Cancer; mALBI, modifed albumin-bilirubin.

**p* <0.05

Data are expressed as number (percentage) or median [interquartile range].

**Table S3. Blood-based predictive markers for the efficacy of atezolizumab plus bevacizumab therapy identified through literature review**

|  | Publication date | Single center/ Multicenter* | Author | Cut-off | Outcome | Reference |
| --- | --- | --- | --- | --- | --- | --- |
| NLR | Oct-21 | Single center | Eso Y | 3.21 | PFS | Curr Oncol. 2021 Oct 14;28(5):4157-4166. |
|  | Jan-22 | Multicenter (a) | Tada T | 3 | OS | Eur J Gastroenterol Hepatol. 2022 Jun 1;34(6):698-706. |
|  | Jan-22 | Single center (b) | Wang JH | 3 | PFS | Cancers (Basel). 2022 Jan 11;14(2):343. |
|  | May-22 | Multicenter | Chuma M | 2.74 | ORR | Hepatol Res. 2022 Mar;52(3):269-280. |
|  | May-22 | Multicenter | Cheon J | 5 | PFS, OS | Liver Int. 2022 Mar;42(3):674-681. |
|  | May-22 | Multicenter (c) | Maesaka K | 3 | HPD | Hepatol Res. 2022 Mar;52(3):298-307. |
|  | Nov-22 | Multicenter | Wu YL | 5 | OS | Cancers (Basel). 2022 Nov 26;14(23):5834. |
|  | Jan-23 | Multicenter | Ochi H | 2.56 | PFS | Hepatol Res. 2023 Jan;53(1):61-71. |
|  | Feb-23 | Multicenter | Chon YE | 2.5 | OS, PFS | Cancer Med. 2023 Feb;12(3):2731-2738. |
|  | Jun-23 | Single center | Jost-Brinkmann F | 3.2 | ORR, PFS | Aliment Pharmacol Ther. 2023 Jun;57(11):1313-1325. |
|  | Nov-23 | Multicenter | Akyildiz A | 2.9 | OS | Medicine (Baltimore). 2023 Nov 10;102(45):e35950. |
| PLR | Jan-22 | Single center (b) | Wang JH | 230 | PFS | Cancers (Basel). 2022 Jan 11;14(2):343. |
| IL-6 | Feb-22 | Multicenter (c) | Myojin Y | 3.2 | PFS, OS | Cancers (Basel). 2022 Feb 10;14(4):883. |
|  | Jan-23 | Multicenter | Yang H | 18.49 | ORR, PFS, OS | JHEP Rep. 2023 Jan 16;5(4):100672. |
| (m)ALBI grade/score | Feb-22 | Multicenter | de Castro T | NA | OS | Ther Adv Med Oncol. 2022 Feb 26;14:17588359221080298. |
|  | Jul-22 | Single center | Tanaka T | 1/2a vs 2b/3 | ORR | Curr Oncol. 2022 Jul 8;29(7):4799-4810. |
|  | Sep-22 | Multicenter (a) | Tanaka T | NA | PFS, OS | Hepatol Res. 2022 Sep;52(9):773-783. |
|  | Nov-22 | Multicenter (d) | Fulgenzi CAM | NA | OS, PFS | Eur J Cancer. 2022 Nov;175:204-213. |
|  | Feb-23 | Single center | Tomonari T | 1/2a vs 2b/3 | ORR, PFS | Cancer Med. 2023 Feb;12(3):2646-2657. |
|  | Jun-23 | Multicenter (e) | Yano Y | 1/2a vs 2b/3 | OS | JGH Open. 2023 Jun 15;7(7):476-481. |
|  | Sep-23 | Multicenter | Takeuchi Y | -2.3 | early PD | Cancer Med. 2023 Sep;12(17):17559-17568. |
|  | Oct-23 | Multicenter | Navadurong H | 1/2a vs 2b/3 | OS | World J Gastrointest Oncol. 2023 Oct 15;15(10):1771-1783. |
| CRAFITY score | Apr-22 | Single center | Teng W | NA | OS, PFS, ORR | Am J Cancer Res. 2022 Apr 15;12(4):1899-1911. |
|  | Oct-22 | Multicenter (a) | Hatanaka T | NA | PFS, OS | Hepatol Int. 2022 Oct;16(5):1150-1160. |
|  | Feb-23 | Single center | Lieb S | NA | OS, PFS | Hepatobiliary Surg Nutr. 2023 Feb 28;12(1):148-150. |
| IGF-1 score | Oct-22 | Multicenter | Kaseb AO | 2 | OS | J Hepatocell Carcinoma. 2022 Oct 11;9:1065-1079. |
| CXCL9 | Oct-22 | Multicenter | Hosoda S | 333 | early PD rate, PFS | Liver Cancer. 2022 Oct 31;12(2):156-170. |
| AFP | Nov-22 | Multicenter (d) | Fulgenzi CAM | 400 | OS | Eur J Cancer. 2022 Nov;175:204-213. |
|  | Oct-23 | Multicenter | Raj R | 400 | ORR | J Gastrointest Surg. 2023 Oct;27(10):2126-2134. |
|  | Jan-24 | Multicenter (c) | Kai M | 400 | OS | PLoS One. 2024 Jan 2;19(1):e0294590. |
| GH | Dec-22 | Single center | Mohamed YI | 3.7/0.9 | OS | Oncotarget. 2022 Dec 6;13:1314-1321. |
| DCP | Feb-23 | Multicenter | Chon YE | 186 | OS, PFS | Cancer Med. 2023 Feb;12(3):2731-2738. |
|  | Jun-23 | Multicenter (e) | Yano Y | 400 | OS | JGH Open. 2023 Jun 15;7(7):476-481. |
| mALF score | Feb-23 | Multicenter (a) | Hatanaka T | NA | OS, PFS | Hepatol Int. 2023 Feb;17(1):86-96. |
| HCC-GRIm score | Feb-23 | Multicenter (a) | Hatanaka T | 3 | OS, PFS | Cancer Med. 2023 Feb;12(4):4259-4269. |
| neo-GPS | Mar-23 | Multicenter (a) | Tada T | NA | OS | Cancer Med. 2023 Mar;12(6):6980-6993. |
| PNI | Apr-23 | Multicenter (a) | Tada T | 47 | OS, PFS | Oncology. 2023;101(4):270-282. |
|  | May-23 | Multicenter (f) | Persano M | 41 | OS, PFS | Oncology. 2023;101(5):283-291. |
| ABE index | Apr-23 | Multicenter (f) | Persano M | NA | OS | Anticancer Res. 2023 Apr;43(4):1599-1610. |
| Osteopontin | Jun-23 | Multicenter | Yamauchi R | 61.375 | PD rate, PFS | J Gastroenterol. 2023 Jun;58(6):565-574. |
| TM score | Aug-23 | Multicenter (a) | Tanaka K | NA | OS, PFS | Cancers (Basel). 2023 Aug 31;15(17):4348. |
| Combination of CRP and NLR | Sep-23 | Multicenter | Ueno M | NA | Any-size reduction, OS | Oncology. 2023;101(9):565-574. |
| GNRI | Oct-23 | Multicenter (a) | Hiraoka A | NA | PFS, OS | Hepatol Res. 2023 Oct;53(10):1031-1042. |
| IP-10/CXCL10 | Oct-23 | Single center | Takada H | 84 | PR rate | Oncology. 2023;101(10):655-663. |
|  | Epub ahead of print | Multicenter | Suzuki T | 690 | OS, PFS | Cancer Med. 2023. doi: 10.1002/cam4.6876. |
| α-FAtE | Mar-24 | Multicenter (f) | Rossari F | NA | OS, TTP | Int J Cancer. 2024;154(6):1043-1056. |
| Eosinophil | Mar-24 | Single center | Toshida K | 170 | ORR, PFS | J Gastroenterol Hepatol. 2024;39(3):576-586. |
| CRP | Epub ahead of print | Multicenter | Kaneko S | 1 | DCR, OS | Hepatol Res. 2023. doi: 10.1111/hepr.14001. |

ABE, atezolizumab plus bevacizumab; AFP, α-fetoprotein; ALBI, albumin-bilirubin; α-FAtE, α-fetoprotein, alkaline phosphatase, eosinophils; CRAFITY, CRP and AFP in immunotherapy; CRP, C-reactive protein; CXCL, chemokine (C-X-C motif) ligand; DCP, des-gamma-carboxy prothrombin; DCR, disease control rate; GH, growth hormone; GNRI, geriatric nutritional risk index; GPS, Glasgow prognostic score; HCC-GRIm, hepatocellular carcinoma modified Gustave Roussy immune; HPD, hyperprogressive disease; IGF, insulin-like growth factor; IL, interleukin; IP, interferon-γ-induced protein; mALBI, modified albumin-bilirubin; mALF, modified albumin-bilirubin grade and α-fetoprotein; NLR, neutrophil-to-lymphocyte ratio; ORR, objective response rate; OS, overall survival; PD, progressive disease; PFS, progression-free survival; PLR, platelet-to-lymphocyte ratio; PNI, prognostic nutritional index; PR, partial response; TM, tumor marker; TTP, time to progression.

*Alphabets within parentheses indicate that multiple predictive markers have been reported by the same institution/group.

**Table S4. Multivariate analysis of the impacts of CRP, AFP, and mALBI grade on progression-free survival in the first-line setting (main analysis cohort^†^)**

| Variables | Hazard ratio | 95% CI | *p* value |
| --- | --- | --- | --- |
| CRP (≥1.0 mg/dL) | 2.33 | 1.32–4.12 | 0.004* |
| AFP (≥100 ng/mL) | 1.52 | 0.98–2.36 | 0.059 |
| mALBI grade (2b/3) | 1.52 | 0.98–2.34 | 0.060 |

BCLC, Barcelona Clinical Liver Cancer; CI, confidence interval; mALBI, modifed albumin-bilirubin.

**p* <0.05

**^†^** This cohort includes patients with a stable disease and a progression-free survival of <180 days.

**Table S5. Subgroup analyses on the impact of CRAFITY score of 2 on the risk of refractoriness in the first-line setting (main analysis cohort^†^)**

| Subgroups | Odds ratio | 95% CI | *p* value |
| --- | --- | --- | --- |
| BCLC stage |  |  |  |
| Early or intermediate stage (*n* = 112) | 2.79 | 0.17–46.1 | 0.473 |
| Advanced stage (*n* = 86) | 6.56 | 1.34–32.1 | 0.020* |
| Macrovascular invasion |  |  |  |
| Absent (*n* = 163) | 9.45 | 1.03–86.8 | 0.047* |
| Present (*n* = 35) | 3.00 | 0.52–17.3 | 0.219 |
| mALBI grade |  |  |  |
| 1/2a (*n* = 131) | 4.41 | 0.70–27.6 | 0.113 |
| 2b/3 (*n* = 67) | 8.00 | 0.94–68.1 | 0.057 |

BCLC, Barcelona Clinical Liver Cancer; CI, confidence interval; mALBI, modifed albumin-bilirubin.

**p* <0.05

**^†^** This cohort includes patients with a stable disease and a progression-free survival of <180 days.

**Table S6. Comparison of the baseline characteristics between the progressive disease and clinical benefit groups in the first-line cohort**

|  | Progressive disease  (*n* = 29) | Clinical benefit  (*n* = 126) | *p* value |
| --- | --- | --- | --- |
| Age, years | 72 [62–79] | 73 [68–78] | 0.654 |
| Sex |  |  | 0.809 |
| Male | 22 (75.9%) | 98 (77.8%) |  |
| Female | 7 (24.1%) | 28 (22.2%) |  |
| Performance status |  |  | 0.285 |
| 0 | 22 (75.9%) | 106 (84.1%) |  |
| 1 | 6 (20.7%) | 18 (14.3%) |  |
| 2 | 1 (3.4%) | 2 (1.6%) |  |
| Body mass index, kg/m^2^ | 23.5 [20.9–24.1] | 24.1 [21.2–26.3] | 0.195 |
| Etiology of liver diseases |  |  | 0.535 |
| Viral | 11 (37.9%) | 58 (46.0%) |  |
| Non-viral | 18 (62.1%) | 68 (54.0%) |  |
| Steatotic liver disease |  |  | 0.679 |
| Presence | 13 (44.8%) | 45 (35.7%) |  |
| Absence | 15 (51.7%) | 75 (59.5%) |  |
| Unknown | 1 (3.4%) | 6 (4.8%) |  |
| Child–Pugh score |  |  | 0.184 |
| 5 | 14 (48.3%) | 74 (58.7%) |  |
| 6 | 10 (34.5%) | 43 (34.1%) |  |
| 7 | 4 (13.8%) | 8 (6.3%) |  |
| 8 | 1 (3.4%) | 1 (0.8%) |  |
| ALBI score | -2.14 [-2.53 to -1.92] | -2.53 [-2.73 to -2.28] | <0.001* |
| mALBI grade |  |  | <0.001* |
| 1 | 5 (17.2%) | 56 (44.4%) |  |
| 2a | 8 (27.6%) | 40 (31.7%) |  |
| 2b | 15 (51.7%) | 30 (23.8%) |  |
| 3 | 1 (3.4%) | 0 (0%) |  |
| BCLC stage |  |  | 0.004* |
| Early stage | 1 (3.4%) | 9 (7.1%) |  |
| Intermediate stage | 9 (31.0%) | 73 (57.9%) |  |
| Advanced stage | 19 (65.5%) | 44 (34.9%) |  |
| Macrovascular invasion | 12 (41.4%) | 14 (11.1%) | <0.001* |
| Extrahepatic metastasis | 8 (27.6%) | 34 (27.0%) | 1.000 |
| NLR | 2.8 [2.3–3.9] | 2.3 [1.7–3.1] | 0.056 |
| PLR | 121 [95–166] | 108 [83–155] | 0.252 |
| CRP, mg/dL | 0.5 [0.2–1.9] | 0.2 [0.1–0.3] | <0.001* |
| AFP, ng/mL | 189 [17–5028] | 8 [4–59] | <0.001* |
| DCP, mAU/mL | 2403 [62–10398] | 213 [49–2470] | 0.039* |

AFP, α-fetoprotein; ALBI, albumin-bilirubin; BCLC, Barcelona Clinical Liver Cancer; CRP, C-reactive protein; DCP, des-gamma-carboxy prothrombin; mALBI, modifed albumin-bilirubin; NLR, neutrophil-to-lymphocyte ratio; PLR, platelet-to-lymphocyte ratio.

Data are expressed as number (percentage) or median [interquartile range].

**p* <0.05

**Table S7. Performance of potential predictors for progressive disease associated with atezolizumab plus bevacizumab therapy in the first-line setting (sensitivity analysis cohort^†^)**

| Predictors | AUROC (95% CI) | Cut-off | Sensitivity | Specificity | PPV |
| --- | --- | --- | --- | --- | --- |
| Continuous values |  |  |  |  |  |
| CRP | 0.766 (0.671–0.861) | ≥1.0 | 31.0% | 92.1% | 9/19 (47.4%) |
| AFP | 0.752 (0.642–0.862) | ≥400 | 44.8% | 84.1% | 13/32 (40.6%) |
| DCP | 0.623 (0.498–0.749) | ≥400 | 58.6% | 59.7% | 17/67 (25.4%) |
| NLR | 0.614 (0.494–0.734) | ≥3.21 | 44.8% | 76.2% | 13/43 (30.2%) |
| PLR | 0.568 (0.451–0.686) | ≥230 | 13.8% | 90.5% | 4/16 (25.0%) |
| ALBI score | 0.702 (0.595–0.809) | ≥-2.3 | 58.6% | 73.8% | 17/50 (34.0%) |
| PNI | 0.702 (0.600–0.803) | ≤47 | 89.7% | 32.5% | 26/111 (23.4%) |
| Categorical values |  |  |  |  |  |
| mALBI grade | 0.695 (0.593–0.797) | 1/2a *vs.* 2b/3 | 55.2% | 76.2% | 16/46 (34.8%) |
| CRAFITY score | 0.722 (0.619–0.825) | 0/1 *vs.* 2 | 27.6% | 97.6% | 8/11 (72.7%) |
| mALF score | 0.741 (0.639–0.843) | 0/1 *vs.* 2 | 41.4% | 93.7% | 12/20 (60.0%) |
| Neo-GPS | 0.690 (0.590–0.791) | 0 *vs.* 1/2 | 82.8% | 42.9% | 24/96 (25.0%) |
| ABE index | 0.643 (0.566–0.720) | Low/Intermediate *vs.* high-risk | 86.2% | 42.1% | 25/98 (25.5%) |
| GNRI | 0.627 (0.514–0.740) | Normal/mild *vs.* moderate/severe | 37.9% | 85.7% | 11/29 (37.9%) |

ABE, atezolizumab plus bevacizumab; AFP, α-fetoprotein; ALBI, albumin-bilirubin; AUROC, area under the receiver operating characteristics curve; CRAFITY, CRP and AFP in immunotherapy; CRP, C-reactive protein; DCP, des-gamma-carboxy prothrombin; GNRI, geriatric nutritional risk index; mALBI, modifed albumin-bilirubin; mALF, modified ALBI grade and AFP; neo-GPS, neo-Glasgow prognostic score; NLR, neutrophil-to-lymphocyte ratio; PLR, platelet-to-lymphocyte ratio; PNI, prognostic nutritional index; PPV, positive predictive value.

**^†^** This cohort excludes patients with a stable disease and a progression-free survival of <180 days.
